# Intraprostatic Botulinum Neurotoxin Type A Injection for Benign Prostatic Hyperplasia—A Spotlight in Reality

**DOI:** 10.3390/toxins8050126

**Published:** 2016-04-26

**Authors:** Yu-Chao Hsu, Hung-Jen Wang, Yao-Chi Chuang

**Affiliations:** 1 Department of Urology, Linko Chang Gung Memorial Hospital, Taipei 24401, Taiwan; yuchaohsu@gmail.com; 2 Department of Urology, Kaohsiung Chang Gung Memorial Hospital, Kaohsiung 83301, Taiwan; hujewang@yahoo.com.tw; 3 College of Medicine, Chang Gung University, Taipei 24401, Taiwan

**Keywords:** botulinum toxin, prostate, lower urinary tract symptoms, benign prostatic hyperplasia

## Abstract

Botulinum toxin is a neurotoxin produced by the bacterium Clostridium botulinum. It inhibits the release of acetylcholine and other neurotransmitters from the nerve terminal. Botulinum toxin, specifically toxin type A (BoNT-A) has been used since the 1970s to reduce the muscular hypercontraction disorders. The application of BoNT-A in urology field started from intra-bladder injection for overactive bladder, which has been recognized as third line therapy in many countries. Since prostate gland as well as bladder is under the influence of autonomic innervation, theorectically, injection of BoNT-A into the prostate induces chemo-denervation and modulation of prostate function, and reduces lower urinary tract symptoms (LUTS). This article reviews the application of BoNT-A in patients with LUTS/ benign prostatic hyperplasia (BPH) from mechanisms of action to clinical results. BoNT-A has been shown to induce prostate apoptosis, downregulation of alpha 1A receptors, and reduce contractile function of prostate in animal studies. Open studies of intraprostate BoNT-A injection have demonstrated promising results of reducing LUTS and improvement of voiding function in human LUTS/BPH, however, intraprostatic BoNT-A injection did not perform better than the placebo group in recent publications of placebo controlled studies. We suggested that BoNT-A prostate injection might benefit selected population of BPH/LUTS, but it is unlikely to be an effective therapy for general population of male LUTS/BPH.

## 1. Introduction

Benign prostatic hyperplasia (BPH) is a disease associated with lower urinary tract symptoms (LUTS). Treatment goal for BPH is to decrease LUTS and release the obstruction; both mechanical obstruction caused by tissue compression due to enlarged prostate, or functional obstruction caused by smooth contraction which was activated via sympathetic alpha-1 adrenoceptors (α1-AR) [1]. It has been widely accepted that α1-AR antagonist with or without 5α reductase inhibitor was considered as the first-line therapy for LUTS suggestive of BPH. Methods for the treatment of BPH have expanded in recent decades. Transurethral resection of prostate, the gold standard for the treatment of medical refractory BPH patients, was challenged by minimally invasive laser intervention, which benefits from lower complications [2]. 

However, not all patients are suitable or willing for operation, and patients with comorbidities may also increase the risks of operation and anesthesia. Some men are willing to accept prostate injection therapy as an office procedure to avoid surgical risks, hospitalization, and lower complications. The concept of intraprostatic injection therapy for BPH has been developed with various results since the early 1900 [3]. Many injectable agents have been explored, with the majority reported to induce coagulative necrosis, followed by shrinkage of prostatic volume, and resulting in improvement of voiding dysfunction. Botulinum toxin A (BoNT-A), a neurotoxin produced by Clostridium botulinum, is regarded as a potent biological toxin for humans. It was known as an agent that blocks the acetylcholine release in presynaptic cholinergic neuromuscular junction and decreases the muscle contractility. BoNT-A was widely used to reduce muscular hypercontractions, such as strabismus, blepharospasm, cerebral palsy, and cosmetic. Recently, the effects of BoNT-A was found to be not only limited to the neuromuscular junction, but also extended into the neuroglandular junction to induce tissue apoptosis and/or decrease muscle contractility without focal inflammatory change. The effect is prompt and has a long duration [4].

BoNT-A injection for treatment of BPH patients has been investigated since 2003 [5]. BoNT-A was injected into transitional zone of prostate under the guidance of transrectal ultrasound or direct injection through cystoscopy. This injection can be done in an office-based procedure with minimal complications. Several pilot studies have demonstrated efficacy of BoNT-A in reducing prostate size and LUTS and the effect was prompt and persisted as long as 12 months [6,7,8,9,10,11]. However, in a phase 2, a placebo controlled study demonstrated sustained and significant improvement in both BoNT-A group and the placebo group without intergroup difference [12]. *Post hoc* analysis suggested a strong placebo effect in intraprostatic injection therapy. Another phase 2 study was designed with sham procedure before the real injection to minimize the placebo effect, and the result showed both onabotulinumtoxinA (Botox, Allergan, NJ, USA) 200 U and placebo improved International Prostate Symptom Score (I-PSS) without group difference [13]. 

Intraprostatic BoNT-A injection inducing the relaxation of the smooth muscle and atrophy and apoptosis of prostate tissue might be effective for BPH/LUTS. We reviewed the mechanisms of action of BoNT-A on the prostate, and update the clinical effects of BoNT-A in the treatment of BPH/LUTS. 

## 2. Material and Methods

We conducted a systemic review of published literature in Pubmed, using Botulinum toxin, prostate, and low urinary tract symptoms as the key words. We focused on effect for low urinary tract symptoms and the safety issue. In total, 64 papers were reviewed and only 48 papers are included. Diagnostic tools included AUA scores, peak urinary flow rate (Q_max_), post-void residual volume (PVR), and ultrasonography confirmed prostate volume (PV). 

The studies were included if they met the following criteria: (1) reported outcome measurements including IPSS, Q_max_, PV, and PVR (2) interventions including administration of BoNT-A; and (3) participants including those diagnosed with LUTS/BPH.

### 2.1. Mechanisms of BoNT-A 

BoNT-A is a 1285 amino acid chain when first synthesized and is activated only when the single chain is cleaved into a 50-kDa light chain and a 100-kDa heavy chain, linked by a single disulphide bond [14]. It blocks the neurotransmission by binding the toxin heavy chain to synaptic vesicle protein SV2. After that, the toxin is internalized to the nerve terminal and the light chain is translocated into the cell cytosol and cleaves the synaptosomal-associated protein (SNAP25), which prevents vesicle fusion with the plasma membrane and inhibits neurotransmitter release.

### 2.2. Effects of BoNT-A 

#### 2.2.1. Motor Effects 

BoNT-A is known to exert paralyzing effects by blocking ACh release from motor nerve. It cleaves the SNAP-25, an essential protein in exocytosis, and prevents the release of ACh in response to nerve impulse. The inhibitory effects of Ach in both somatic and autonomic nerve system are well documented. Intramuscular injection of BoNT-A can achieve temporary chemo-denervation and make both skeletal and smooth muscle relaxation. 

#### 2.2.2. Sensory Effects

Some studies show evidence to support that BoNT-A might inhibit afferent neurotransmission and achieve analgesic effect [15]. It has been demonstrated that BoNT-A inhibits the release of calcitonin gene-related peptide (CGRP), substance P, glutamate, nerve growth factor(NGF), and ATP [15,16], which are all sensory mediators. Furthermore, BoNT-A pretreatment was shown to inhibit COX-2 expression in the prostate and spinal cord in a capsaicin induced prostatitis model, demonstrating that BoNT-A can suppress central sensitization [17].

#### 2.2.3. Glandular Effects

BoNT-A influences the morphology and secretory function by inhibiting the soluble *N*-ethylmaleimide-sensitive fusion protein attachment protein receptors (SNAREs) of acini cells in rat salivary glands [18]. Teymoortash *et al.* showed clear signs of glandular atrophy after application of BoNT-A in glandular cells. Functional change with less electron dense, smaller size, and polymorph are also noted when compared to control group. The authors suggested that these effects may be due to glandular denervation induced by the inhibition of the SNAREs involved in acetylcholine release at the neuroglandular junction and also specially inhibition of those involved in exocytosis of the granula of the acinar cells. 

### 2.3. Rational for BoNT-A Injection in Prostate

The prostate tissue is rich in adrenergic and muscarinic receptors thus the function of the prostate is significantly influenced by autonomic nerve. Cholinergic innervation by parasympathetic nerve plays an important role in the growth and secretion of prostate epithelium while noradrenergic innervation by sympathetic nerve controls the contraction of smooth muscle and is one of the etiology of outflow obstruction accompanying LUTS/BPH [1,19]. Furthermore, sympathetic stimulation induces epidermal growth factor and has a trophic function in prostate growth [20]. BoNT-A acts as an chemo-denervation agent may block the release of neurotransmitters and modulate the autonomic nerve function, and may have a therapeutic effect on patients with LUTS/BPH. 

In animal models, atrophy and apoptosis of prostate glandular tissue was demonstrated in rat prostate injected with BoNT-A [21,22]. However, the function of the prostate is not only influenced by acetylcholine but also other factors such as androgen, and noradrenaline [1,19,21]. Interestingly, the alpha 1-A adrenergic receptor was also decreased after BoNT-A injection. However, the androgen receptor has no change. 

The dog is one of a few animals that develop BPH spontaneously. Lin and colleagues demonstrated injection of onabotulinumtoxinA 200 U into the dog prostate decreases the urethra pressure under the stimulation of norepinephrine and electrostimulation when compared with the normal saline injection group. However, the result was not seen on onabotulinumtoxinA 100 U group. In histology study, prostate tissue after onabotulinumtoxinA 200 U injection showed more glandular atrophy than onabotulinumtoxinA 100 U injection group [23]. This study suggested that onabotulinumtoxinA 200 U may exert effect on both static components and dynamic components of BPH. 

### 2.4. Technique for Prostate Injection 

Prostatic injections of BoNT-A can be performed using transperineal, transurethral, or transrectal approaches. Transrectal prostatic injection is the most familiar procedure for urologists just like transrectal prostate needle biopsy. However, transperineal echo guide injection minimized the complication of infection seen in the transrectal approach. Transurethral injection using a cystoscopically-adapted needle provides a direct view of the prostate, but regional or general anesthesia is needed. The transurethral approach facilitates the management of trilobular prostate enlargement. 

Therapeutic doses of BoNT-A have been reported from 100 units to 300 units of OnabotulinumtoxinA in volumes ranging from 4 cc to 20 cc. OnabotulinumtoxinA 200 U is typically used and reconstituted with 4 mL of normal saline or approximately 10% of total prostate volume. Two to four points of injection are carried on to each side of prostate. A 21–23 gauge 15 or 20 cm long needle is used under the guidance of transrectal ultrasound. Two mL of BoNT-A is injected into each side or into two separate spots in each lateral lobe. Diffusion of hyperechoic BoNT-A over the lateral lobe of the prostate can be easily seen with transrectal sonographic monitoring [1].

### 2.5. Clinical Data

BoNT-A intra-prostatic injection was first reported in 2003 by Maria *et al.* in 30 patients with a markedly enlarged prostate and a uroflow rate not more than 15 mL/s [5]. In BoNT-A (200 U, onabotulinumtoxinA) treated patients significant increases in maximum flow rates from 8.1 to 16.8 mL/s (52%) as well as significant decreases in post void residual (PVR) from 126.3 to 21.0 mL (83%) were seen. Prostate volumes decreased from 52.6 to 16.8 mL (68%), Prostate specific antigen (PSA) levels dropped from 3.7 to 1.8 ng/mL (51%) and an improvement of the American Urological Association (AUA) symptom scores from 23.2 to 8.0 (65%) was noted. Signs and symptoms were stable through 6 and 12 months in the BoNT-A treated patients.

Kuo *et al.* injected the onabotulinumtoxinA 200 U via a cystoscopy in patients with urinary retention or high residual urine and poor surgical candidates. All 10 patients had improvement in spontaneous voiding, 8 reported excellent result, and 2 felt improved. The total prostate size decrease from 65.6 to 49.5 mL (29.9%) and the peak flow rate increased as well. Both voiding pressure and postvoid residual urine volume were significantly decreased [7]. 

Silva *et al.* reported 21 men with BPH on chronic indwelling catheter for at least three months who were unfit for surgery received 200 U onabotulinumtoxinA in the transition zone by the transrectal approach [9]. Baseline prostate volume of 70 ± 10 mL decreased to 57 ± 10 mL at one month, and to 47 ± 7 mL at three months. At one month, 16 patients (76%) could resume voiding with a mean Q_max_ of 9.0 ± 1.2 mL/s. At three months, 17 patients (81%) voided with a mean Q_max_ of 10.3 ± 1.4 mL/s. Residual urine was less than 100 mL at the two time points. Furthermore, mean serum total PSA decreased from 6.0 ± 1.1 ng/mL at baseline to 5.0 ± 0.9 ng/mL at three months.

Chuang *et al.* [8]**,** a total of 41 men (mean age 69.1 ± 7.1 years) with an IPSS ≥ 8, peak flow rate less than 12 mL/s and refractory to medical treatment were injected with 100 U (*N =* 21, for prostate volume less than 30 mL) or 200 U (*N =* 20, for prostate volume greater than 30 mL) of onabotulinumtoxinA into the prostate transperineally under transrectal ultrasound guidance. Thirty one out of 41 patients (75.6%) have more than 30% improvement on LUTS and quality of life (QOL) indices [8,10]. Twelve of 41 patients (29.2%) did not have change of prostate volume; however, 7 out of the 12 patients (58.3%) still have more than 30% improvement in maximal flow rate, LUTS, and QOL.

As seen from our previous review article of application of botulinum toxin in the prostate [24], there are many small clinical studies that report symptomatic relief and improvement in the measured parameters during the follow-up period, whereas local or systematic side-effects are rare. Although the results of the clinical studies are encouraging, the level of evidence is low from these open studies. 

Crawford *et al.* conducted the first multicenter, double-blind, randomized phase II clinical trial of onabotulinumtoxinA to treat the BPH/LUTS [25]. Men over 50 years old diagnosed as BPH, with an American Urological Association(AUA) symptom index greater than eight, peak flow rate less than 15 mL/s, voiding volume 125 mL or greater, and a post-void residual 350 mL or less were enrolled for injection of 100 U or 300 U onabotulinumtoxinA. In the 100 unit arm (*N =* 68) the mean baseline AUA symptom index of 18.8 decreased by 7.1 and 6.9 at 3 and 12 months, respectively. In the 300 unit arm (*N =* 66) the baseline of 19.5 decreased by 8.9 and 7.1, respectively. In the 100 unit arm the mean baseline maximum urinary flow rate of 10.0 mL per second increased by 2.5 and 2.2, respectively, and in the 300 unit arm the baseline of 9.6 increased by 2.6 and 2.3, respectively. Both the 100 U and 300 U groups met the primary outcome for treatment efficacy and safety with no significant difference. 

The study was not designed to compare the effect of onabotulinumtoxinA to placebo or as a dose ranging study with a placebo control group. The authors suggested that there is a substantial placebo effect in the medical treatment of BPH which may be exaggerated by injection therapy. Marberger *et al.* conducted a double-blind randomized, placebo-controlled study for men older than 50 years who were diagnosed as BPH/LUTS with (IPSS) ≥ 12, total prostate volume (TPV) 30–100 mL, and Q_max_ 5–15 mL/s [12]. Single transperineal (*N =* 63) or transrectal (*N =* 311) administration of placebo (*N =* 94) or onabotulinumtoxinA 100 U (*N =* 95), 200 U (*N =* 94), or 300 U (*N =* 97) into the prostate transition zone.

Significant improvements from the baseline in IPSS, Q_max_, TPV, and TZV were observed for all groups, including placebo, at week 12 (*p <* 0.001), with no significant differences between onabotulinumtoxinA and placebo. However, in an exploratory *post hoc* analysis, a significant reduction in IPSS *vs.* placebo was observed with onabotulinumtoxinA 200 U in prior α-blocker users (*N =* 180) at week 12. 

McVary *et al.* designed a multicenter, double-blind, randomized, placebo controlled parallel group trial [13]. Patients with an I-PSS 14 or greater, peak urinary flow rate 4 to 15 mL per second, and a total prostate volume 30 to 80 mL were randomized 1:1 to a single intraprostatic treatment of onabotulinumtoxinA 200 U or placebo. A single-blind sham procedure followed by a four-week run-in was included to attempt to minimize any potential placebo effect. Patients who still met eligibility criteria after the run-in entered the double-blind active treatment period. Of 427 patients enrolled 315 were randomized and treated. Decreases from baseline in I-PSS were observed in the onabotulinumtoxinA and placebo groups (−6.3 *vs*. −5.6 points, *p* < 0.001) with no difference between the groups overall or in subgroups. Improvement was observed in the peak urinary flow rate, which was significant only at week six compared to placebo. Improvement was significant at all time-points in a patient subgroup on stable concurrent α-blockers or 5α-reductase inhibitors during the study. They concluded that onabotulinumtoxinA 200 U and placebo improved I-PSS score with no difference. The reason for placebo effect is still unclear. 

The ideal candidates for receiving minimally invasive treatment for BPH/LUTS are patients who are refractory to oral medication, and do not want operation due to potential surgical complications or who are poor candidates for operation. The first RCT by Marberger *et al.* included 50% without any previous oral medication for BPH/LUTS [12]. The McVary study included 75% of patients who, at the moment of inclusion, had abandoned oral BPH/LUTS treatment [13]. An exploratory *post hoc* analysis of the subgroup of patients who had received alpha-blockers (180 out of 380, 47%) showed a significant reduction from baseline in IPSS in the onabotulinumtoxinA 200-U group *versus* placebo in Marberger study. Furthermore, in the McVary trial, the sub-analysis in the population on concurrent BPH medical therapy showed an improvement in Q_max_ that was significantly greater in the onabotulinumtoxinA arm than in the placebo arm. Taken together, onabotulinumtoxinA injection for BPH/LUTS, is influenced by the selected population.

Robert *et al.* [26] conducted a randomized clinical trial comparing prostatic injection of BoNT-A (Botox^®^; tans-rectal injections of 200 U in 10 cc saline; *N =* 64) to optimized medical therapy (*N =* 63) in the treatment of LUTS related to BPH. Thirty days (D30) after randomization BoNT-A patients were asked to stop any medical therapy related to LUTS. In the control group medical treatment could be adapted until D30 but was not changed from D30 to D120. One month after BoNT-A injection, 73.3% of these patients could interrupt their medical treatment and the effects of BoNT-A prostatic injection on IPSS (from 16.9 ± 7.2 to 12.0 ± 6.7) was not inferior to optimized medical treatment (from 15.7 ± 7.3 to 11.8 ± 6.9) on D120. After adjustment for baseline IPSS, delta IPSS between study groups at D120 was 0.04; IC95% [−2.16; 2.09] [26].

Shim *et al.* conducted a systematic review and meta-analysis of the published literature in PubMed, Cochrane Library, and Embase reporting on onabotulinumtoxinA use in LUTS/BPH [27]. Single-group analysis for the placebo effect and meta-regression analysis for the moderator effect were performed with three high-quality RCTs [5,12,13] compared with placebo.

A total sample size of 522 subjects (260 subjects in the experimental group with 200 U onabotulinumtoxinA and 262 subjects in the control group). Study duration ranged from 8 to 24 weeks. The pooled overall standardized mean differences in the mean change in IPSS for the onabotulinumtoxinA group *versus* the placebo group was −1.02 (95 % CI −1.97, −0.07). The other outcomes (Q_max_, prostate volume, and post-voided residual volume) were not statistically different between the two groups. The placebo effect in single-group analysis ranged from 0% to 27.9% for IPSS, and from −1.1% to 28.7% for Q_max_ (lowest to highest, respectively). The results of this study do not provide evidence of clinical benefits of using the onabotulinumtoxinA injection for LUTS/BPH in real clinical practice. The authors concluded that the placebo effects might be overlooked in the previous open studies, and led to overestimation of BoNT-A effects.

Men with LUTS/BPH may have exaggerated placebo response to prostate injection therapy. However, BoNT-A-induced chemodenervation, and inhibited the release of acetylcholine and noradrenaline have been well documented. Theroretically, chemodenervation should also have significant effects on the prostate. Nevertheless, there was no study to show the current injection methods can achieve real prostate-BoNT-A interaction. How much of the BoNT-A solution leaked out of prostate? Previous publications have demonstrated that 80% of patients displayed some evidence of extravasation and 35% of patients had significant extravasation and a mean of 17.6% volume was found in the extraperitoneal fat tissue after intravesical BoNT-A injection [28,29]. It is an important issue to prove which injection method can provide sufficient duration of prostate BoNT-A interaction before we can conclude that BoNT-A has no therapeutic effects on the LUTS/BPH.

## 3. Summary

Intraprostatic injection of BoNT-A, as a minimally invasive therapy for LUTS associated with benign prostatic hyperplasia, seems attractive with certain rationality, but so far, available clinical results with a high level of evidence are lacking. The result that BoNT-A is not better than placebo suggests that BoNT-A is unlikely to be a therapy for the population of male LUTS/BPH through the current injection methods. However, BoNT-A prostate injection might benefit from a selected population of LUTS/BPH. 
